# The Effect of Sex Peptide and Calorie Intake on Fecundity in Female *Drosophila melanogaster*

**DOI:** 10.1100/tsw.2009.126

**Published:** 2009-10-14

**Authors:** Blanka Rogina

**Affiliations:** Department of Genetics and Developmental Biology, School of Medicine, University of Connecticut Health Center, Farmington, CT, USA

**Keywords:** sex peptide, Drosophila melanogaster, reproduction, egg laying, calorie restriction

## Abstract

The accessory gland proteins (Acps) of the male Drosophila cause changes in the behavior and physiology of female flies. Sex peptide (SP) is one of the Acps that initiates many changes, including an increase in egg production. The data presented here show that SP overexpression in transgenic (G-10) female flies increases egg production when females are kept on a standard and high-calorie diet, relative to controls that do not express SP. Particularly, a high increase in egg production observed in G-10 females on a high-calorie diet suggests that SP overexpression magnifies the female response to caloric uptake. However, on a calorie-restricted diet, the fecundity of G-10 females overexpressing SP is lower than control females. On a high-calorie diet, mating increases early egg production in G-10 and control females, but lifelong total egg production is only increased in control females, most likely due to the physiological changes set off by substantial initial egg production in G-10 females.

### INTRODUCTION

Mating in *Drosophila melanogaster* is a complex process, extending beyond a brief physical interaction between males and females to a disparate set of physiological consequences observed in female flies. A negative relationship between reproduction and longevity has been reported for both sexes in a number of experimental animals. *Drosophila* virgin females and males live longer than those that are mated[1,2,3,4,5,6]. However, the effect of mating on survivorship and fecundity varies with the age of the female fly[6]. Mating early in life causes dramatic increases in egg production and female mortality rate, but if males are removed, an initial increase in mortality is partially reversed. During midlife, however, mating does not increase female egg production, but does cause an irreversible increase in mortality rate[6]. Although mating decreases male survivorship as well, females experience many other physiological changes that modify their behavior. Postmating changes in female physiology are caused by the 112 male accessory gland proteins (Acps) that are transferred to females during mating[7]. These proteins and peptides increase the rate of egg production and egg laying, regulate proteolysis, affect immune response, reduce female receptivity to further mating, and decrease survivorship[7,8,9,10]. Acps also affect transfer and storage of the sperm, and displace sperm from previous matings.

Sex peptide (SP) (Acp70A) was one of the first isolated Acps and its physiological role has been the object of numerous investigations[11,12]. Injection of isolated SP into virgin females is sufficient to prevent mating and increase egg production[11]. Similarly, overexpression of SP in transgenic female flies decreases their receptivity to matings and causes extrusion of the ovipositor, while absence of SP causes only a weak and short response to mating[9,12,13]. SP stimulates the corpora allata, endocrine glands, to secrete lipid-like juvenile hormone (JH), which stimulates oogenesis and vitellogenesis[14]. SP also affects the expression of a limited number of genes, increases food intake, and alters immune response and antimicrobial peptide synthesis[15,16,17,18].

Similar to reproduction, the level of caloric uptake profoundly affects the physiology and survivorship of a variety of species, including flies. Calorie restriction (CR), defined as a decrease in caloric uptake without starvation, decreases reproduction and weight, delays age-related pathophysiology, and increases mobility and survivorship[19,20,21,22,23]. The goal of this investigation was to determine how the overexpression of SP affects egg production when females are kept on different calorie levels. The flies used in experiments were transgenic G-10 flies, in which the SP gene is continuously and ectopically overexpressed in the adult fat body by use of the promoter of a yolk protein enhancer[12]. Thus, the G-10 females continuously synthesize SP in the fat body and secrete into the hemolymph. The data presented here show that SP overexpression in females increases egg production when females are kept on standard and high-calorie diets, but decreases production under CR. Mating increases early egg production in females on a high-calorie diet, but has no effect on lifelong total egg production in SP-overexpressing females, most likely due to physiological changes that occur when energy resources are shifted from egg production to deal with the effects of mating and Acps.

### METHODS

#### Dietary Calorie Content of *Drosophila* Food

Standard laboratory corn media, as well as food marked as 0.5X, 1.0X, and 3.0X, were used. The three food levels are standardized. 1.0X is the food that has 100 g/l of sucrose (MP Biomedicals), 100 g/l of brewer's yeast (MP Biomedicals), and 20 g/l of agar[19,20]. 0.5X food contains 50 g/l of sucrose and 50 g/l brewer's yeast, and 3.0X food contains 300 g/l sucrose and 300 g/l brewer's yeast, and both have 20 g/l agar.

#### Fly Stocks

The G-10 (transformant line G-10-3) and *cn, ry*$^{42}$ strains were generously provided by Drs. Eric Kubli, Stephen L. Helfand, and Claudio Pikienly[12]. The G-10 flies express SP constitutively in their fat bodies from where SP is released into the hemolymph. The line was originally generated in Kubli's laboratory and described in detail by Aigaki et al.[12]. Briefly, the flies were generated by germline transformation of the *cn, ry*$^{42}$ strain with construct containing yolk protein (*yp1)* promoter fused to the *SP* gene; *yp1* drives ectopic expression of SP in the fat body of G-10 flies. Flies were maintained in a humidified temperature-controlled environmental chamber at 25°C (Percival Scientific) on a 12-h light:dark cycle.

#### Fertility Studies

Virgin flies were collected on $CO_2$. Single virgin females, or a single female and a single male of the same age, were kept in a plastic vial containing a 0.5X, 1.0X, or 3.0X food medium at 25°C in a humidified incubator. Dead males were replaced with the sibling males of the same age. The flies were passed to new vials daily, and the number of eggs and age of death for each individual female were recorded.

### Statistical Analysis

Generalized estimating equations (GEEs) using STATA 10.0 statistical software were used to test the effects of genotype (G-10 vs. *cn, ry*), food levels (0.5X, 1.0X, and 3.0X), and age[24,25]. GEEs were also used to determine whether G-10 virgins on 3.0X food produced more or less eggs than *cn*, *ry* virgins on 3.0X food over their lifetime. Using a traditional linear model, such as the analysis of variance (ANOVA), would have been inappropriate for these data since the distribution of egg production was heavily positively skewed with many instances of zero egg production. The negative binomial link was used as the appropriate link function.

## RESULTS

It has been previously reported that injecting or overexpressing SP in female *Drosophila* increases their egg laying and suppresses their receptivity to further mating[12]. It is known that the caloric content of the food also greatly affects female fecundity. In order to examine the interaction of these two major modifiers on *Drosophila* fecundity, the daily egg production of female transgenic flies overexpressing SP (G-10) and genetically matched control (*cn, ry*) flies maintained on food with three different caloric contents were compared. Overexpression of SP ectopically in fat bodies of G-10 flies is driven by yolk protein enhancer (*yp1*)[12]. The parental flies were grown on standard corn laboratory media and on the day of eclosion, a single male and female were placed in a vial with food of one of the three calorie levels: 1.0X, 3.0X, and 0.5X, where 1.0X resembles standard laboratory food, but does not contain corn[19,20]. The 0.5X food has a 50% lower caloric content than 1.0X, and is considered CR food. Transgenic flies overexpressing SP had a 75% increase in the average egg production on 1.0X food levels compared to the genetic control (G-10 = 182.2; *cn, ry* = 104.0) (Fig. 1A, Table 1). The number of eggs laid by the control and experimental flies was lower than the number observed in *Canton-S* or $w^{1118}$ wild-type stocks previously reported, presumably due to their genetic background[20,23,26,27]. In order to maximize egg production, we kept G-10 and *cn, ry* flies on very high-calorie food, 3.0X. G-10 females on 3.0X food laid 100.1% more eggs compared to 1.0X food levels (1.0X = 182, 3.0X = 364.5) (Fig. 1D). However, *cn, ry* flies laid only 15.7% more eggs on 3.0X compared to 1.0X food (1.0X = 104, 3.0X = 120) (Fig. 1E). Thus, a total of 302% increase in egg production was observed in G-10 females compared to *cn, ry* females on 3.0X food (Fig. 1B, Table 1). GEE analysis reported that a statistically significant three-way age × group × food interaction was found ($p < 0.001$) (Supplemental Table 1), i.e., G-10 flies laid more eggs than controls on 1.0X and 3.0X food. In addition, flies of the same genotype laid more eggs on 3.0X compared to 1.0X food.

### G-10 Females Overexpressing SP are more Vulnerable to CR

We determined the egg laying of G-10 and control flies on a low-calorie diet, 0.5X. Control females *cn, ry* laid 34.0% fewer eggs on 0.5X food compared to 1.0X food (1.0X = 104, 0.5X = 68.6) (Fig. 1C–E, Table 1). G-10 flies on 0.5X food had 89% lower average lifelong egg production compared to the G-10 flies on 1.0X and, surprisingly, 70.1% lower compared to control flies on 0.5X food (0.5X: G-10 = 20; *cn, ry* = 68.6) (Fig. 1C–E, Table 1). It is possible that although these females had increased fecundity compared to the control flies on a normal or high-calorie diet, additional effects of SP made flies more vulnerable to the stressful condition of CR.

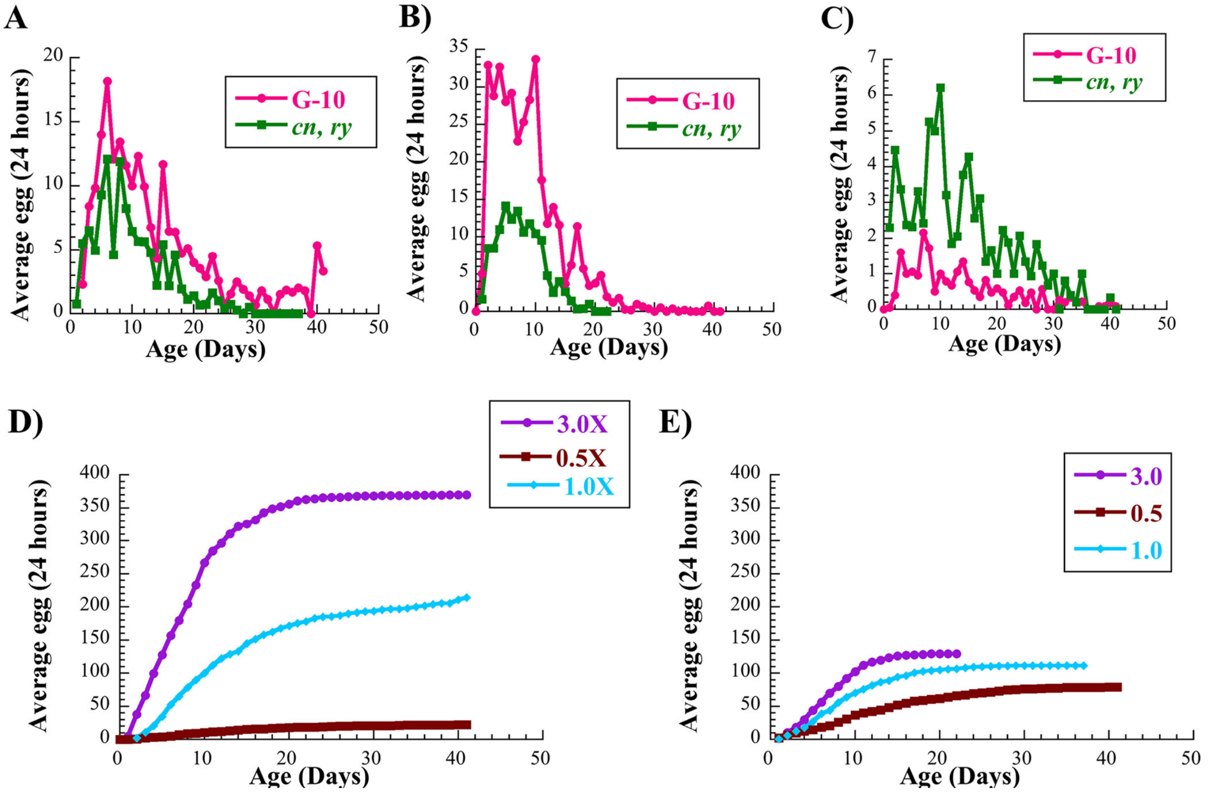

**FIGURE 1.** Overexpression of SP increases the fecundity of females on 1.0X and 3.0X food levels. Daily egg production of G-10 flies overexpressing SP (magenta) and control *cn, ry* flies (green) on 1.0X (A), 3.0X (B), and 0.5X food (C). (D and E) Cumulative egg production of G-10 female flies (D) and genetic controls, *cn,ry* (E) on 3.0X (purple), 1.0X (blue), and 0.5X (brown) food levels presented in A, B, and C. G-10 flies have higher average and cumulative lifelong egg production on 1.0X and 3.0X food, but laid fewer eggs on 0.5X food. There were 20 females in each experiment.

**TABLE 1**
**SP Increases the Egg Production in Females on High and Normal Food Levels**

| Group | Food Levels | Average Lifelong Number of Eggs/Fly | SE Eggs/Fly | Average Eggs/Day | SE Average Eggs/Day |
|---|---|---|---|---|---|
| G-10 | 3.0 | 364.5 | 28.9 | 8.82 | 2.6 |
| *cn, ry* | 3.0 | 120.3 | 18.1 | 5.86 | 1.1 |
| G-10 | 1.0 | 182.2 | 22.9 | 4.48 | 1.1 |
| *cn, ry* | 1.0 | 104.0 | 15.3 | 3.0 | 0.7 |
| G-10 | 0.5 | 20.0 | 3.6 | 0.42 | 0.1 |
| *cn, ry* | 0.5 | 68.6 | 10 | 1.47 | 0.4 |
| G-10, virgins | 3.0 | 385.2 | 33.1 | 8.0 | 2.1 |
| *cn, ry,* virgins | 3.0 | 80.3 | 12.3 | 2.71 | 0.6 |

*Note*: G-10 females produce more eggs during their life with the exception of females on 0.5X food compared to the *cn, ry* controls. Females were mated from day 0 or kept as virgins when marked. There were 20 female flies in each experiment. SE = standard error.

### G-10 Overexpression Appears to Magnify the Fly's Response to Different Food Levels

Fig. 1B indicates that female flies on 3.0X food laid most of their eggs during the first 10 days of life. Females on the 1.0X food produced fewer eggs during their lifetime compared to 3.0X, however, they produced eggs longer in life. In order to further examine how egg-laying patterns changed with the age of G-10 and *cn, ry* females, we compared the sum of eggs laid in a 10-day period by females on different food levels (Fig. 2A and B, Table 2). The G-10 flies exhibited a much more pronounced difference than control flies in the number of eggs laid on different food levels in the 10-day period, demonstrating that not only do G-10 flies produce more eggs, but they also respond more to different food levels. G-10 flies produced 134.5% more eggs on 3.0X food compared to 1.0X food during the first period, while *cn, ry* flies produced only 42.6 % more on 3.0X compared to 1.0X food (G-10: 3.0X = 233, 1.0X = 99.4; *cn, ry*: 3.0X = 100.3, 1.0X = 70.3) (Fig. 2A and B, Table 2). Similarly, control flies exhibited a greater decline in the eggs laid from one time period to another than did the G-10 flies on the same food levels. For instance, G-10 flies on the 3.0X food level produced 49 and 89% fewer eggs in the second and the third periods, respectively, compared to the first period, while *cn, ry* flies had 80% fewer eggs produced during the second period and no eggs laid in the third period. Negative binomial regressions found a statistically significant increase in eggs laid by G-10 females compared to *cn, ry* females on 1.0X food during the first three periods and on 3.0X during the first two periods. G-10 females laid significantly fewer eggs than *cn, ry* females on 0.5X food during the first three periods (Supplemental Tables 2–4).

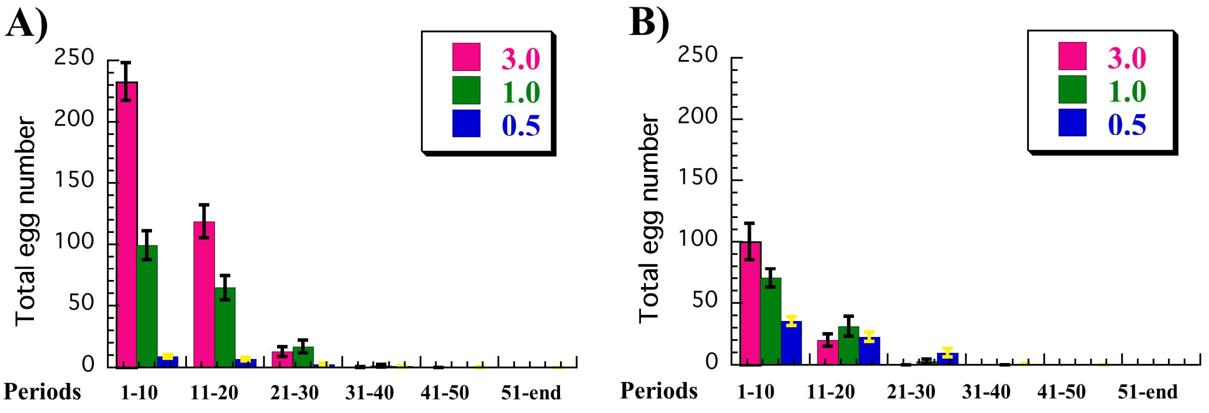

**FIGURE 2.** Overexpression of SP increases female response to different caloric food levels. Average total number of eggs laid by G-10 flies overexpressing SP (A) and genetic controls, *cn, ry* (B) kept on 3.0 X (magenta), 1.0X (green), or 0.5X (blue) food levels during 10-day periods. Periods are expressed in days. There is a bigger difference in the total number of eggs per period laid by G-10 compared to control females on 1.0X and 3.0X food. However, G-10 females laid fewer eggs on 0.5X food compared to *cn, ry* females. There were 20 females in each experiment.

### SP Overexpression Modifies Female Response to Mating

Virgin G-10 females overexpress SP, which increases their egg production. Mated G-10 females receive additional SP and the other Acps provided by males during mating, which affects female fecundity as well. In order to find out how different levels of SP affect egg laying, lifelong egg production was determined in females overexpressing SP and control females, both of which were kept as virgins or mated from day 0. Since female fecundity is the highest on a very high-calorie diet, cumulative effects would be most easily detected on this type of food. Thus, females were kept on the 3.0X food level. Mating changed both the total number and age-specific egg-laying pattern (Fig. 3A and B, Table 2). As expected, the number of eggs laid by G-10 virgin females was significantly higher than the number of

**TABLE 2**
**SP Increases Average Egg Laying in Females during 10-Day Periods**

| Genotype | Food Level | Eggs (1–10) | Eggs (11–20) | Eggs (21–30) | Eggs (31–40) | Eggs (41–50) | Eggs (51–End) |
|---|---|---|---|---|---|---|---|
| G-10 | 3.0X | 233 (15.4) | 118.2 (13.4) | 12.98(3.8) | 0.45 (0.3) | 0 | |
| *cn, ry* | 3.0X | 100.25 (14.9) | 20.05 (4.8) | 0 | | | |
| G-10 | 1.0X | 99.35 (11.7) | 64.55 (9.7) | 17.0 (5.2) | 1.45 (1.0) | 0 | |
| *cn, ry* | 1.0X | 70.3 (7.5) | 31.2 (8.0) | 2.85 (1.8) | 0 | | |
| G-10 | 0.5X | 9 (1.4) | 6.85 (1.4) | 2.75 (0.9) | 1.00 (0.4) | 0.50 (0.3) | 0 |
| *cn, ry* | 0.5X | 35.3 (3.5) | 22.7 (3.9) | 9.85 (3.3) | 0.65 (0.4) | 0.1 (0.1) | 0 |
| G-10 | 3.0X virgins | 149 (12.2) | 172.45 (14.6) | 58.85(9.7) | 4.2 (1.2) | 0.4 (0.4) | 0.15 (0.2) |
| *cn, ry* | 3.0X virgins | 33.5 (6.2) | 42.3 (8.6) | 4.55 (1.6) | 0 | | |

*Note*: Average egg laying for flies overexpressing SP (G-10) and genetic control (*cn, ry*) flies during 10-day periods: 0–10, 21–30, 31–40, 41–50, and from 51 days until the end. Females were mated from day 0 or kept as virgins. Standard errors of the average eggs laid in a 10-day period are included in parenthesis. There were 20 females in each experiment.

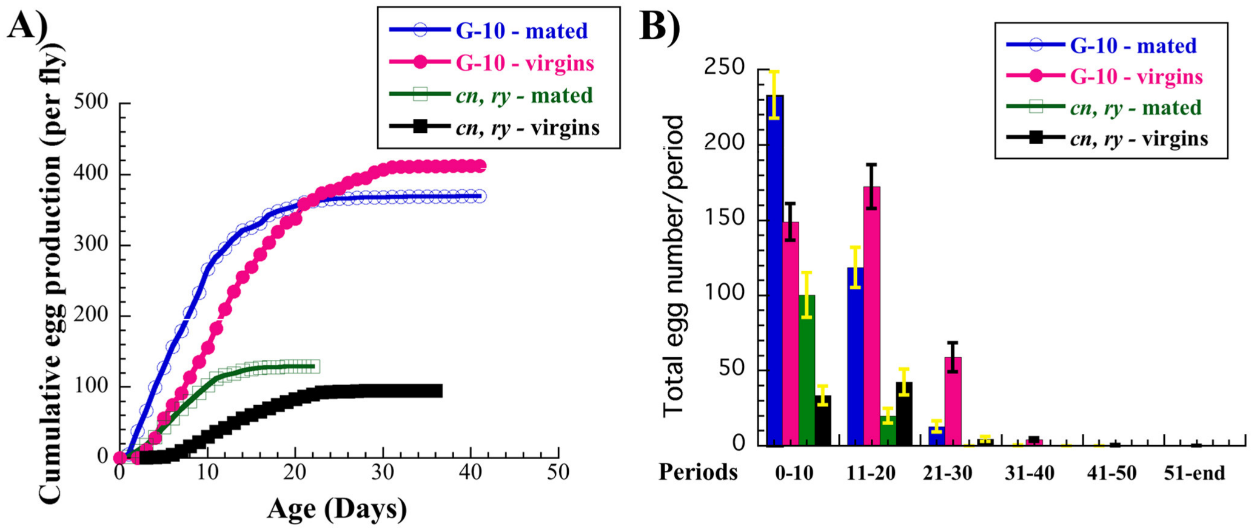

**FIGURE 3.** Mating increases early egg laying in G-10 flies overexpressing SP. (A) Cumulative lifelong egg production in G-10 flies overexpressing SP (blue and magenta)and genetic controls, *cn, ry* (green and black) mated from day 0 (blue and green) or kept as virgins (magenta and black). Female flies produced more eggs early when mated from day 0 compared to virgins, but the total number of eggs in mated G-10 is lower compared to the virgins. (B) Total egg number produced by G-10 (blue and magenta) and *cn, ry* (green and black) females kept as mated (blue and green) or virgins (magenta and black) in 10-day periods. Mated females produced more eggs during the first 0- to 10-day period, while virgin females laid the most eggs during the second period from 11 to 20 days. There were 20 females in each experiment.

eggs laid by *cn, ry* females (G-10 = 385.2; *cn, ry* = 80.3; z = 7.47; $p < 0.001$) (Tables 1 and 2, Supplemental Tables 5–7).The total lifelong number of eggs in mated control *cn, ry* females is 33.3% higher than that of virgin females (mated = 120.3; virgins = 80.3; z = –5.08; $p < 0.001$). However, the total number of eggs produced in mated G-10 females was 5% lower compared to the virgin G-10 flies (G-10: mated = 364.5; virgins = 385.2). During the first 10 days of adult life, both G-10 and control females had the greatest increase in egg laying in response to mating. Mated G-10 and *cn, ry* flies laid 56 and 199% more eggs during the first 10 days after mating compared to the virgin female of the same genotype, respectively (Fig. 2B, Table 2). The effect of mating on egg production was less pronounced in

G-10 flies since they already overexpressed SP, but they still laid more eggs compared to the control *cn, ry,* flies. However, mated G-10 and *cn,ry* females produced fewer eggs from 11 to 20 days of age. G-10 produced 31% and *cn, ry* produced 52% fewer eggs. A similar trend of decreased egg production was observed until the end of life of mated females. Conversely, virgin G-10 and *cn, ry* females laid more eggs during the second period compared to the first (Fig. 3B, Table 2). There are significant differences in age-dependent patterns of egg laying between mated and virgin females in both G-10 and *cn, ry* (Supplemental Tables 6 and 7).These data suggest that a combination of SP overexpression and transfer of Apcs during mating increases egg production early in life, but has no additional effect on female fecundity. In fact, the mated G-10 females laid fewer eggs during their lifetime.

### DISCUSSION

SP is one of the major components produced by the male accessory glands. SP and other Acps get transmitted during mating into the female reproductive organs[9]. The amino terminal end of SP binds to sperm, which act as SP carriers during sperm and seminal fluid transfer. After mating, sperm and SP are stored in spermathechae and seminal receptacles for more then a week. SP can then be slowly released from sperm and facilitate prolonged effects by binding with its carboxy terminal end to the sex peptide receptor (SPR)[15,28]. The expression of SPR is widespread, but the number of primary targets of SP is relatively small; only six to eight sensory neurons located in the uterus that coexpressed *pickpocket* (*ppk*) and fruitless (*fru*)[29,30]. The gene *ppk* encodes a sodium channel subunit that has a role in mechanosensation; *fru* is the male-specific transcription factor, which is produced by alternative splicing of the transcript. SP mediates a postmating response in females by binding to the SPR and by the neurotransmission of that signal to the CNS[30]. Binding of SP to SPR affects the physiology and behavior of the female in several ways: it increases egg production and egg laying, decreases the receptivity of females to further mating, increases food intake, and initiates an immune response. It has been shown previously that injection of SP or overexpressing of SP in transgenic female flies increases egg production and decreases the receptivity of females to mating[12].

Another means to affect female fecundity is by modulating caloric uptake. A number of reports show that although caloric content of the food is important, the ratio between sugar and yeast has a larger effect on survivorship and reproduction[23]. We have previously reported that varying caloric content of the food from 0.5X to 3.0X, but keeping the yeast:sugar ratio the same, results in a dramatic increase in egg laying[20]. Here, three different food levels were used to assess the difference in egg production between females overexpressing SP and controls. Overexpression of SP in the fat body is driven by the yolk protein (*yp1)* enhancer[12]. SP gets secreted from the fat body into the hemolymph and can then reach its target SPRs. As expected, transgenic females overexpressing SP (G-10) laid 75% more eggs during their life on 1.0X food compared to the controls. On high-calorie 3.0X food, G-10 females produced and layed twice as many eggs as they did on 1.0X food. Interestingly, there was only a modest increase of 15% in the egg production observed in control females kept on 1.0X vs. 3.0X food. Thus, the total difference in eggs laid between SP-overexpressing and control flies on 3.0X food is threefold. Part of the huge increase in egg production observed in G-10 females compared to controls on 3.0X is due to increased food consumption caused by SP[16]. Mated females and females overexpressing SP consume more food than females kept as virgins or females mated with males that do not have SP[16]. Microarray analysis of the abdomens and heads of females overexpressing SP showed increased expression of genes involved in metabolism several hours after mating[17]. This metabolic switch is most likely necessary for increased egg production. Consequently, the G-10 females on 3.0X produced and laid the most eggs.

Flies kept on 0.5X food experienced many beneficial effects identical to those described in other species on CR. For instance, flies on 0.5X food had decreased weight, increased spontaneous locomotor activity, changes in biochemistry, and increased survivorship[20,23,31]. The effect of caloric uptake on reproduction has been widely reported. Females on CR suppress their fertility until the conditions improve. Once the food resources become available, reproduction is resumed. One unexpected observation was decreased egg production in G-10 females relative to controls on 0.5X food medium. While the level of egg production was decreased in control females, 34% on low-calorie food levels relative to 1.0X food, G-10 females performed much worse.

### Mating does not Further Increase the Egg Production in G-10 Females

Mated control females produced more eggs in comparison to the control females kept as virgins on 3.0X food. The total lifelong number of eggs in mated control females is 33.3% higher compared to the virgin controls. Interestingly, mating can still increase early egg production in G-10 females, most likely due to the transfer of additional Acps that promote egg production, such as ovulin and AD99. The prohormone ovulin (ASP26Aa) affects egg laying in females during the first day after mating by increasing ovulation[32]. DUP99B, made in the ejaculary duct, increases ovulation and oviposition similarly to SP[33].

In the experiments reported here, mated G-10 females laid significantly more eggs during the first 10 days of life compared to the virgin G-10 females. However, after the first 10 days, egg production in mated G-10 females diminished relative to virgins to the extent that the total lifelong egg production of the mated females was 5% less than the virgins. One explanation for the dramatic mating-induced lowering of egg production after the first 10 days after mating could be a physiological change to keep the balance between the reproduction, body maintenance, and DNA repair. That is, during the first 10-day period after eclosion, most of the energy is diverted to the extraordinary production of eggs and to combat the toxic effect of the mating itself, including the harmful effect of Acps on female physiology. During the second time period, there may be less available energy, which has to be shifted from egg production to be used for somatic maintenance and to deal with the effect of mating. We have previously reported that female *Drosophila* adjust their egg production to cope with age-associated changes and increased frailty[6]. A model of how the calorie content of the food may affect the balance between energy storage, reproduction, and somatic maintenance has been described[23]. The data presented here extend those findings by examining the relationships between caloric uptake, mating, and SP overexpression on egg production and show a dramatic effect when high-caloric intake was combined with SP overexpression. The lifelong pattern of egg production suggest a physiological shift to divert the available energy resources from a high-energy expenditure for egg production and egg laying, to somatic maintenance and DNA repair in order to deal with the harmful effects of mating and the toxic effect of Acps on female physiology.

### ACKNOWLEDGMENTS

I thank Suzanne Kowalski for excellent technical support, Drs. Joseph Jack and Stewart Frankel for critically reading the manuscript, and Drs. Kubli and Aigaki for helpful information and fly stocks. I am grateful to Dr. Daniel J. Denis for expert statistical analysis. This work was supported by grant from the National Institute on Health RO1AG023088.

## SUPPLEMENT

**SUPPLEMENTAL TABLE 1**
**GEE Model of Egg Count as a Function of Age, Group, and Food**

| Source | Coefficient | Std. Error | z | P>\|z\| | 95% Conf. Interval |
|---|---|---|---|---|---|
| Constant | –0.2242927 | 0.0265221 | –8.46 | 0.000 | –0.2763, –0.1723 |
| Age | –0.0179105 | 0.0029022 | –6.17 | 0.000 | –0.0236, –0.0122 |
| Group (G10)$^{+}$ | –0.0279054 | 0.0091025 | –3.07 | 0.002 | –0.0457, –0.0101 |
| Food (1.0X)$^{++}$ | 0.180883 | 0.0272304 | 6.64 | 0.000 | 0.1275, 0.2343 |
| Food (3.0X)$^{++}$ | 0.3824739 | 0.0268578 | 14.24 | 0.000 | 0.3298, 0.4351 |
| Age × group (G-10) | –0.0119449 | 0.0050691 | –2.36 | 0.018 | –0.0219, –0.0020 |
| Age × food (1.0X) | 0.0043898 | 0.0034637 | 1.27 | 0.205 | –0.0024, 0.0112 |
| Age × food (3.0X) | –0.0299225 | 0.0040751 | –7.34 | 0.000 | –0.0379, –0.0219 |
| Age × group (G-10) × food (1.0X) | 0.0152823 | 0.0055898 | 2.73 | 0.006 | 0.0043, 0.0262 |
| Age × group (G-10) × food (3.0X) | 0.0302861 | 0.0059664 | 5.08 | 0.000 | 0.0186, 0.0420 |

$^{+}$*cn, ry* is used as reference group; "group" consists of mated *cn, ry* vs. G-10; $^{++}$Food = 0.5 g is used as reference group.

The model is statistically significant [Wald $\chi^2(9) = 861.40$, $p < 0.001$]. There is evidence of a three-way day × group × food interaction [z = 2.73, $p < 0.001$; z = 5.08, $p < 0.001$]. What this means is that the two-way day × group interaction depends on what level of food the flies were kept. Hence, the three-way interactions represent differences between two-way interaction effects. There is also an age effect [z = –6.17, $p < 0.001$], a group effect [z = –3.07, $p < 0.001$], and a food effect [z = 6.64, $p < 0.001$; z = 14.24, $p < 0.001$].

### Negative Binomial Regressions

The generalized estimating equation found evidence for a three-way interaction, age (days) × group × food. To tease apart the three-way interaction, we perform negative binomial regressions at each of the age-period factors (1–10, 11–20, 21–30, 31–40, 41–55 days) for each of the food levels and genotypes.

**SUPPLEMENTAL TABLE 2**
**Egg Count as a Function of Group (*cn, ry* vs. G-10), Age, Food = 1.0X**

| Age (Days) | Group | | Coefficient | Std. Error | z | P>\|z\| | 95% Conf. Interval |
|---|---|---|---|---|---|---|---|
| | *cn, ry* | G-10 | | | | | |
| 1–10 | 70.3 | 99.35 | 0.35 | 0.131 | 2.68** | 0.007 | 0.0944, 0.6074 |
| 11–20 | 31.2 | 64.55 | 0.68 | 0.148 | 4.61*** | 0.000 | 0.3918, 0.9714 |
| 21–30 | 2.85 | 17.0 | 1.24 | 0.371 | 3.33*** | 0.001 | 0.5107, 1.9667 |
| 31–40 | 0 | 1.45 | 16.09 | 1246.307 | 0.01 (ns) | 0.990 | –2426.629, 2458.807 |
| 41–55 | 0 | 0 | — | — | — | — | — |

"ns", denotes not statistically significant; *denotes $p < 0.05$; **denotes $p < 0.01$; ***denotes $p < 0.001$.

**SUPPLEMENTAL TABLE 3**
**Egg Count as a Function of Group (*cn, ry* vs. G-10), Age, Food = 3.0X**

| Age (Days) | Group | | Coefficient | Std. Error | z | P>\|z\| | 95% Conf. Interval |
|---|---|---|---|---|---|---|---|
| | *cn, ry* | G-10 | | | | | |
| 1–10 | 100.25 | 233 | 0.823 | 0.121 | 6.79*** | 0.000 | 0.586, 1.061 |
| 11–20 | 20.05 | 118.2 | 1.227 | 0.173 | 7.10*** | 0.000 | 0.888, 1.566 |
| 21–30 | 0 | 12.98 | 18.427 | 3059.719 | 0.01 (ns) | 0.995 | –5978.513, 6015.367 |
| 31–40 | 0 | 0 | — | — | — | — | — |
| 41–55 | 0 | 0 | — | — | — | — | — |

“ns”, denotes not statistically significant; *denotes $p < 0.05$; **denotes $p < 0.01$; ***denotes $p < 0.001$.

**SUPPLEMENTAL TABLE 4**
**Egg Count as a Function of Group (*cn, ry* vs. G-10), Age, Food = 0.5X**

| Age (Days) | Group | | Coefficient | Std. Error | z | P>\|z\| | 95% Conf. Interval |
|---|---|---|---|---|---|---|---|
| | *cn, ry* | G-10 | | | | | |
| 1–10 | 35.3 | 9 | –1.38 | 0.126 | –10.96*** | 0.000 | –1.622, –1.130 |
| 11–20 | 22.7 | 6.85 | –1.16 | 0.133 | –8.70*** | 0.000 | –1.417, –0.896 |
| 21–30 | 9.85 | 2.75 | –1.51 | 0.225 | –6.71*** | 0.000 | –1.953, –1.070 |
| 31–40 | 0.62 | 1.0 | –0.71 | 0.446 | –1.51 (ns) | 0.130 | –1.619, 0.209 |
| 41–55 | 0.1 | 0.5 | 0.80 | 1.212 | 0.66 (ns) | 0.508 | –1.574, 3.177 |

“ns”, denotes not statistically significant; *denotes $p < 0.05$; **denotes $p < 0.01$; ***denotes $p < 0.001$.

**SUPPLEMENTAL TABLE 5**
**Egg Count as a Function of Group (*cn, ry* Virgin vs. G-10 Virgin), Age, Food = 3.0X**

| Age (Days) | Group | | Coefficient | Std. Error | z | P>\|z\| | 95% Conf. Interval |
|---|---|---|---|---|---|---|---|
| | *cn, ry* | G-10 | | | | | |
| 1–10 | 33.5 | 149 | 1.511 | 0.179 | 8.46*** | 0.000 | 1.161, 1.861 |
| 11–20 | 42.3 | 172.45 | 1.270 | 0.099 | 12.84*** | 0.000 | 1.076, 1.464 |
| 21–30 | 4.55 | 58.85 | 1.693 | 0.209 | 8.08*** | 0.000 | 1.282, 2.103 |
| 31–40 | 0 | 4.2 | 0.737 | 0.285 | 2.58 | 0.010 | 0.178, 1.296 |
| 41–55 | 0 | 0.4 | 8.061 | 1801.367 | 0.00 | 0.996 | –3522.553, 3538.676 |

“ns”, denotes not statistically significant; *denotes $p < 0.05$; **denotes $p < 0.01$; ***denotes $p < 0.001$.

#### SUPPLEMENTAL TABLE 6
### Egg Count as a Function of Age and Group (*cn, ry* vs. G-10, vs. *cn, ry* Virgins, vs. G-10 Virgins) Kept on 3.0X Food

| Source | Coefficient | Std. Error | z | P>\|z\| | 95% Conf. Interval |
|---|---|---|---|---|---|
| Constant | –0.058516 | 0.0112206 | –5.22 | 0.000 | –0.0805, –0.0365 |
| Day | –0.0097498 | 0.0017459 | –5.58 | 0.000 | –0.0132, –0.0063 |
| gd1 (G-10)+ | 0.1295281 | 0.0118999 | 10.88 | 0.000 | 0.1062, 0.1529 |
| gd2 (*cn, ry* virgins)+ | –0.1595707 | 0.0313958 | –5.08 | 0.000 | –0.2211, –0.0980 |
| gd3 (G-10 virgins)+ | 0.0848104 | 0.0113509 | 7.47 | 0.000 | 0.0626, 0.1071 |
| Age × gd1 | –0.0106837 | 0.0021409 | –4.99 | 0.000 | –0.0149, –0.0065 |
| Age × gd2 | 0.0071629 | 0.0023118 | 3.10 | 0.002 | 0.0026, 0.0117 |
| Day × gd3 | –0.0018271 | 0.0019604 | –0.93 | 0.351 | –0.0057, 0.0020 |

+Reference group is *cn, ry*; the model is egg count = day + group (four levels), all on 3.0X food.

#### SUPPLEMENTAL TABLE 7
### Egg Count as a Function of Age and Group (*cn, ry* vs. G-10, vs. *cn, ry* Virgins, vs. G-10 Virgins) Kept on 3.0X Food (Using *cn, ry* Virgins as Reference Group)

| Eggs | Coefficient | Std. Error | z | P>\|z\| | 95% Conf. Interval |
|---|---|---|---|---|---|
| Constant | –0.2180865 | 0.029323 | –7.44 | 0.000 | –0.2755586, –0.1606144 |
| Age | –0.002587 | 0.0015153 | 1.71 | 0.088 | –0.0055568, 0.0003829 |
| gd11 | 0.1595706 | 0.0313965 | 5.08 | 0.000 | 0.0980345, 0.221106 |
| gd22 | 0.2890983 | 0.0295896 | 9.77 | 0.000 | 2311037, 0.3470928 |
| gd33 | 0.2443806 | 0.0293731 | 8.32 | 0.000 | 0.1868103, 0.3019509 |
| Age gd11 | –0.0071629 | 0.0023118 | –3.10 | 0.002 | –0.0116939, –0.002632 |
| Age gd22 | –0.0178466 | 0.0019573 | –9.12 | 0.000 | –0.0216829, –0.0140102 |
| Age gd33 | –0.00899 | 0.0017581 | –5.11 | 0.000 | –0.0124358, –0.0055442 |

gd11 = *cn, ry;* gd22 = G-10; gd33 = G-10 virgins.
